# A Pyrene‐Triazacyclononane Anchor Affords High Operational Stability for CO_2_RR by a CNT‐Supported Histidine‐Tagged CODH

**DOI:** 10.1002/anie.202117212

**Published:** 2022-03-23

**Authors:** Umberto Contaldo, Mathieu Curtil, Julien Pérard, Christine Cavazza, Alan Le Goff

**Affiliations:** ^1^ Univ. Grenoble Alpes, CNRS, DCM 38000 Grenoble France; ^2^ Univ. Grenoble Alpes, CEA, CNRS, IRIG, CBM 38000 Grenoble France

**Keywords:** Azamacrocycles, CO_2_ Reduction, Carbon Monoxide Dehydrogenase, Carbon Nanotubes, Pyrene

## Abstract

An original 1‐acetato‐4‐(1‐pyrenyl)‐1,4,7‐triazacyclononane (AcPyTACN) was synthesized for the immobilization of a His‐tagged recombinant CODH from *Rhodospirillum rubrum* (*Rr*CODH) on carbon‐nanotube electrodes. The strong binding of the enzyme at the Ni‐AcPyTACN complex affords a high current density of 4.9 mA cm^−2^ towards electroenzymatic CO_2_ reduction and a high stability of more than 6×10^6^ TON when integrated on a gas‐diffusion bioelectrode.

## Graphical abstract

A pyrene‐triazacyclononane‐Ni carbon‐nanotube anchor affords million‐turnover stability for an electroenzymatic CO_2_‐to‐CO reduction reaction by a recombinant carbon monoxide dehydrogenase.

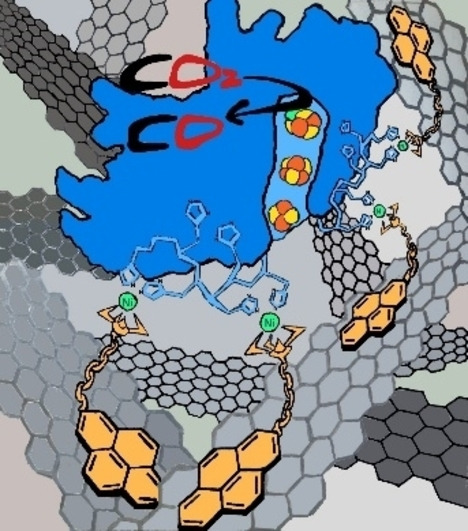

## Introduction

**Figure 1:**
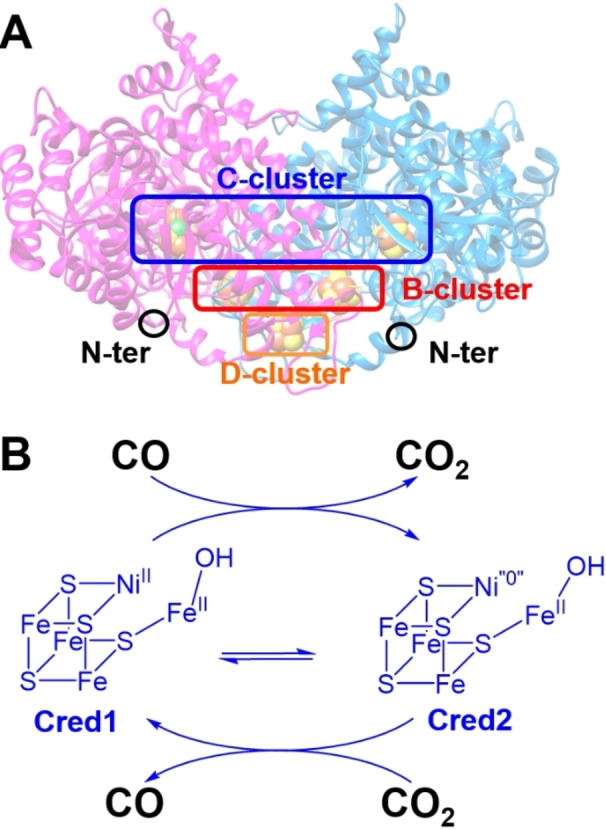
A) Schematic representation of *Rr*CODH dimer indicating the C‐cluster (NiFe_4_S_4_ cluster, active site), B‐clusters (intramolecular Fe_4_S_4_ clusters), D‐cluster (intermolecular Fe_4_S_4_ cluster) and N‐terminal positions. B) Simplified mechanism for the reversible CO_2_ reduction at the C‐cluster involving Cred1 and Cred2 states.

Great efforts have been devoted to synthesize catalysts for the CO_2_ reduction reaction (CO_2_RR) into valuable chemicals or fuels such as CO, HCOOH or CH_3_OH with 100 % selectivity. In particular, synthesizing rare‐metal‐free catalysts for the CO_2_‐to‐CO reduction reaction is intended to participate to the next generation of cost‐effective and industrially‐viable CO_2_ electrolyzers.[^1^, ^2^, ^3^, ^4^, ^5^, ^6^] For this purpose, many efficient molecular catalysts have been recently developed based on iron, nickel, molybdenum or cobalt complexes.[^2^, ^7^, ^8^, ^9^, ^10^, ^11^] However, many of these catalysts possess substantial overpotentials towards CO_2_RR, associated with competing H_2_ evolution and, often, designed for operation in organic solvents, at high temperature or in alkaline media. In nature, Ni‐dependent carbon monoxide dehydrogenase (CODH) is responsible for the reversible reduction of CO_2_ to CO and the concomitant biosynthesis of chemicals such as ethanol, acetate, methane or formate (Figure 1). Owing to billions of years of evolution, CODH achieves the CO_2_RR with high catalytic activity, at near neutral pHs and with minimal overpotential.[^12^, ^13^, ^14^, ^15^, ^16^, ^17^] However, their inactivation by O_2_ is a limiting factor for their use in operational devices either for CO oxidation or CO_2_ reduction.^[12]^ Studies of CO oxidation in the presence of O_2_ have demonstrated that an inactive state is formed which can only be partially reactivated. Furthermore, this reactivation is highly dependent on the nature of the CODH and its mechanism is mostly unknown.[^13^, ^18^, ^19^] Competitive inhibition of CO_2_RR by oxygen is also a major challenge in the design of CO_2_RR catalysts. O_2_ can either directly inhibit catalysts, or its reduction into H_2_O_2_ can compete with CO_2_ reduction or produce highly reactive species such as hydroxide radicals, especially when the catalyst used does not meet the minimal overpotential requirement.^[20]^ The high catalytic efficiency of CODH has been further evidenced when the enzyme is immobilized at the surface of an electrode with CODH behaving as a reversible electrocatalyst towards both electroenzymatic CO_2_RR and CO oxidation.

We have recently developed the engineering of one of the most efficient CODHs towards CO_2_RR.^[16]^

The recombinant CODH from *Rhodospirillum rubrum* (Rec‐*Rr*CODH) was combined with functionalized carbon‐nanotube‐based electrodes, achieving high performance towards CO_2_RR. However, the immobilization of this Rec‐*Rr*CODH relied on hydrophobic interactions owing to a Direct‐Electron‐Transfer (DET) promoter, 1‐pyrenebutyric acid adamantyl amide. Thus, stability up to 8×10^5^ TON was observed over one hour.

The immobilization of biomolecules on surfaces and nanomaterials via specific interactions is a powerful tool in bioanalytical sciences.^[21]^ An effective approach is to use a variety of tags that can be easily and reproducibly introduced at the surface, or at the terminal positions of biomolecules, without any loss of their biological activity. Such immobilization strategies are mostly based on affinity or supramolecular interactions, favoring self‐assembly of the bioentities on surfaces. These strategies provide many advantages over covalent chemistry or using polymers since biological activity is barely affected by the immobilization process. One of the most well‐known approaches is the modification of surfaces with the chelating ligand nitrilotriacetic acid (NTA) followed by the complexation of a divalent metal ion such as Cu^2+^, Ni^2+^ or Co^2+^.[^21^, ^22^] Such metal complexes have a strong affinity towards poly‐histidine sequences and this tool is largely used in protein analysis, notably for the purification of histidine‐tagged enzymes. This strategy was therefore successfully adapted at the surface of electrodes for biosensor and biofuel cell applications[^21^, ^23^, ^24^, ^25^] through the synthesis of pyrene‐NTA (PyNTA) molecules.^[26]^ Pyrene is a soft and stable way of functionalizing CNT sidewalls by π‐π interactions between pyrene and graphene walls for the immobilization of enzymes.[^27^, ^28^, ^29^, ^30^, ^31^, ^32^] This type of bifunctional molecule allows the subsequent immobilization of biomolecules via the Ni‐NTA anchoring group. Recently L. Martin and colleagues developed the use of a self‐assembled monolayer on gold using a thiol modified with a 1‐acetato‐4‐benzyl‐triazacyclononane, (Acbztacn) moiety.^[33]^ This moiety possesses a similar 4‐atom metal‐binding site, but with an increase in the stabilization of the metal/ligand interaction via a macrocycle effect.[^33^, ^34^, ^35^] This type of ligand, whose synthesis has been previously developed by Spiccia et al.^[36]^ have shown superior selectivity and stability towards the immobilization of simple redox His‐tagged proteins such as thioredoxin, plastocyanin and Green Fluorescent Protein.^[33]^ In this work, we synthesized an original 1‐acetato‐4‐(1‐pyrenyl)‐1,4,7‐triazacyclononane (AcPyTACN). We took advantage of this TACN moiety for the immobilization of His‐tagged redox enzymes at CNT sidewalls. We demonstrate the efficient functionalization of CNTs via the AcPyTACN and the realization of a highly‐stable low overpotential CO_2_RR at gas‐diffusion bioelectrodes, accompanied with improved oxygen tolerance.

## Results and Discussion

**Figure 2:**
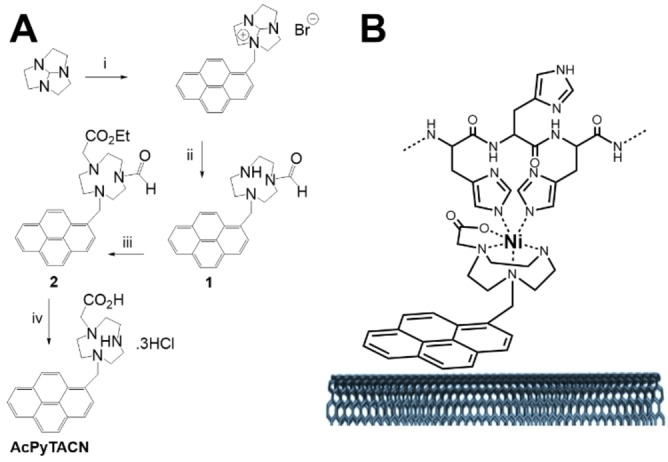
A) Synthesis of AcPyTACN: i) BrCH_2_pyrene/THF; ii) NaOH; iii) BrCH_2_CO_2_Et/Na_2_CO_3_/CH_3_CN; iv) HCl/reflux; B) Ni complexes formed from the MWCNT sidewalls modified with AcPyTACN for the binding of histidine tagged enzymes.

AcPyTACN was synthesized as a hydrochloride salt in 4 steps starting from 1,4,7 triazatricyclo[5.2.1.0^4,10^]decane (Figure 2).

This starting derivative was prepared from the commercially available 1,4,7‐triazacyclononane as previously described.[^37^, ^38^] This derivative reacts with 1‐pyrenylmethyl bromide in THF affording the corresponding monoamidinium bromide salt. Hydrolysis of this derivative gave access to the formyl derivative (**1**) in 87 % yield. Reaction of **1** with ethylbromoacetate in MeCN yielded the ester derivative **2** in 63 % yield after chromatography. This product was characterized by ^1^H NMR and ^13^C NMR. Characteristic ^1^H NMR signals (Figure S1) confirm the presence of pyrenyl, formyl and ester residues. As many compounds bearing the TACN moiety, this derivative exists as two conformational isomers in a two‐third/one‐third ratio, arising from a rotation allowed around the C−N amide bond.[^36^, ^39^] This product was further deprotected (removal of formyl and ester groups) by reflux in 5 M HCl, giving access to the final **AcPyTACN** zwitterionic derivative as a highly insoluble green solid.

**Figure 3:**
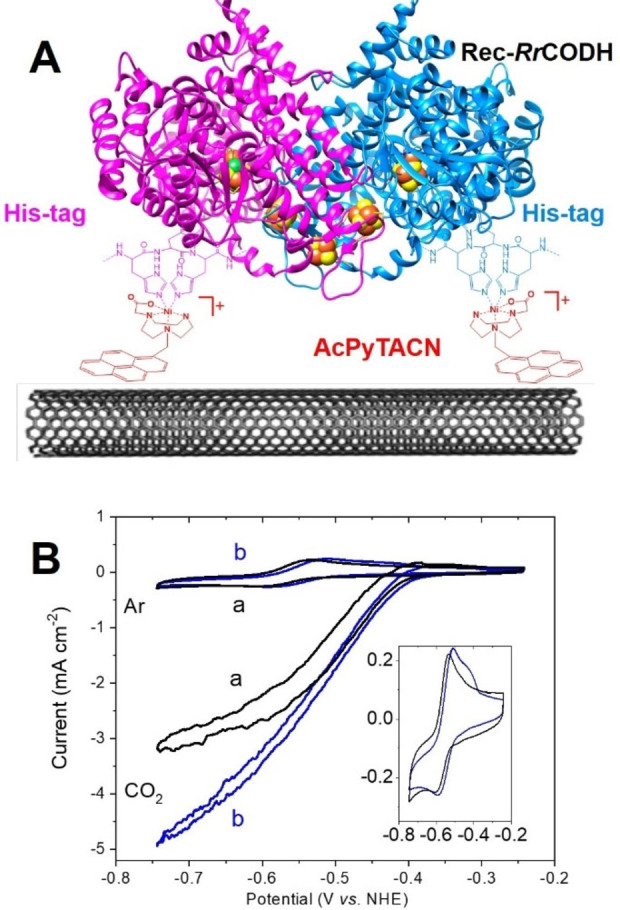
A) Schematic representation of AcPyTACN‐modified MWCNT for the immobilization of Rec‐RrCODH^His^. B) CVs of Rec‐RrCODH^His^‐functionalized (a, black) pristine MWCNT electrode and (b, blue) Ni‐AcPyTACN‐modified MWCNT electrodes under Ar (inset) and CO_2_ (50 mM Tris‐HCl, pH 8.5, *v*=5 mV s^−1^).

The His‐tagged Rec‐*Rr*CODH (Rec‐*Rr*CODH^His^) was immobilized at MWCNT electrodes modified with Ni‐AcPyTACN according to Figure 3A. MWCNT were functionalized by successive incubation steps with the pyrene solution in DMF and a solution of NiCl_2_ in water to form the corresponding monocationic Ni^II^ complex on the CNT surface. The MWCNT electrodes were finally modified by incubation of 38 μM (dimer) of Rec‐*Rr*CODH^His^ for 4 hours. Electrochemistry of pristine MWCNT and AcPyTACN‐functionalized MWCNT electrodes were compared under Ar and CO_2_ at pH 8.5 (Figure 3).

A reversible redox system is observed at *E*_p1/2_=−0.59 V vs. NHE for all electrodes, corresponding to the *C*_red1_/*C*_red2_ bioelectronic redox couple. It is noteworthy that a partial contribution to the current response is likely attributed to the reduction of traces of CO_2_ in the experimental buffer (as well as subsequent CO oxidation formed in the MWCNT layer). The use of cyanide allows the inhibition of this partial catalytic contribution and the estimation of surface coverage. High concentration of cyanide (2 mM) leads to the fully‐inhibited Cred1‐CN adduct, previously characterized by EPR studies.^[16]^ This system, which cannot be reduced into Cred2, as confirmed by EPR titration,^[16]^ is responsible for the irreversible system observed in Figure S2. From the integration of this peak, surface coverages, Γ_max_, of 21 (+/−4) and 47 (+/−4) pmol cm^−2^ were respectively measured for nonmodified MWCNT and **AcPyTACN**‐functionalized MWCNT electrodes according to the irreversible redox system observed in the presence of 2 mM cyanide (Figure S2). It is noteworthy that these values take into account the monomer (instead of the homodimer) surface concentrations, corresponding to the number of active sites per cm^−2^.

ICP‐AES quantification was performed in order to compare Ni and Fe contents at these MWCNT electrodes (see Table S1 in Supporting Information). According to the analysis of the Fe content, higher amounts of CODH are immobilized on **AcPyTACN**‐functionalized MWCNT electrodes, exhibiting an increase in immobilized enzymes of 35 %, corroborating results observed by CV under Ar and CO_2_. As expected, **Ni‐AcPyTACN**‐functionalized MWCNT electrodes exhibit high Ni/Fe ratio, indicating that there is approximatively one *Rr*‐CODH per 25 Ni‐AcPyTACN complexes. Under CO_2_, an irreversible electrocatalytic wave is observed for all electrodes with a half‐wave potential of *E*_red_=−0.54 V vs. NHE. No electrocatalytic response was observed for the **AcPyTACN**‐functionalized MWCNT electrode after Ni^II^ binding, without immobilized CODH (Figure S3). A maximum current density of 4.9 (+/−0.2) mA cm^−2^ was measured for **AcPyTACN**‐functionalized MWCNT electrodes while nonmodified MWCNT electrodes exhibited a maximum current density of 3.1 (+/−0.2) mA cm^−2^. All current densities are given considering the geometrical surface of the MWCNT‐modified electrode (0.07 cm^−2^). These results show the superior performances of Ni‐**AcPyTACN** to immobilize Rec‐*Rr*CODH^His^, underlining the fact that the His‐tag is involved in the immobilization of the enzyme on electrodes. As previously investigated, the fact that MWCNT electrodes exhibit fairly high electrocatalytic activity mostly arises from excellent hydrophobic interactions between CODH and MWCNTs. Experiments were also performed on CODH after removal of the His‐tag (Figure S4). Surprisingly, electrocatalytic performances, as well as catalyst loadings, exhibit very similar performances with maximum current density of 4.8 mA cm^−2^ for the **AcPyTACN**‐functionalized MWCNT electrode. When closely looking at the N‐terminal amino‐acid sequence of *Rr*CODH (where the histidine tag is introduced), the presence of three histidines and two cysteines in the first ten residues might be involved in the binding of the N‐terminal domain at the Ni‐**AcPyTACN** sites of the electrode. These results underline the ability of these sites to accommodate histidine‐rich domains and further confirms their efficiency in enzyme immobilization. It is noteworthy that similar redox signals under argon, as well as a similar sigmoidal shape of the electrocatalytic waves under CO_2_ is indicative that AcPyTACN does not provide better statistical orientation of immobilized enzymes over pristine MWCNTs. Electrochemical data under Ar, CO or CO_2_ do not provide further evidence of a minimization of tunneling distances between the electrode and internal redox centres owing to the **Ni‐AcPyTACN** anchorage point since no difference in distribution of heterogeneous electron transfer rates nor in CO_2_RR overpotentials suggest a significant difference in enzyme orientation.[^40^, ^41^, ^42^] According to crystallographic data from *Rr*CODH structure (PDB: 1JQK), minimal distances between D, B and C clusters and the protein surface are 6.5, 12.1 and 13.1 Å respectively. The N‐terminal amino acid bearing the histidine tag is located at 18.4, 12.4 and 20.2 Å from the D, B and C clusters, respectively. These distances show that all clusters are at distances which are relevant in respect to biological tunneling distances[^43^, ^44^] either from the surface of the protein or from the N‐terminal and considering the fact that the poly‐histidine peptidic chain is highly flexible. Since we previously observed that *Rr*CODH is efficiently adsorbed at hydrophobic surfaces.^[16]^ and considering the fact that hydrophobic patches, as well as the histidine tags are both located near the D‐ and B‐cluster, we can hypothesized that there is a contribution of both to the favorable immobilization of the enzyme during the incubation process. The presence of the Ni‐AcPyTACN moiety likely provides a further improvement in the increase of the enzyme surface coverage during this step without significantly decrease electron tunneling distances.

**Figure 4:**
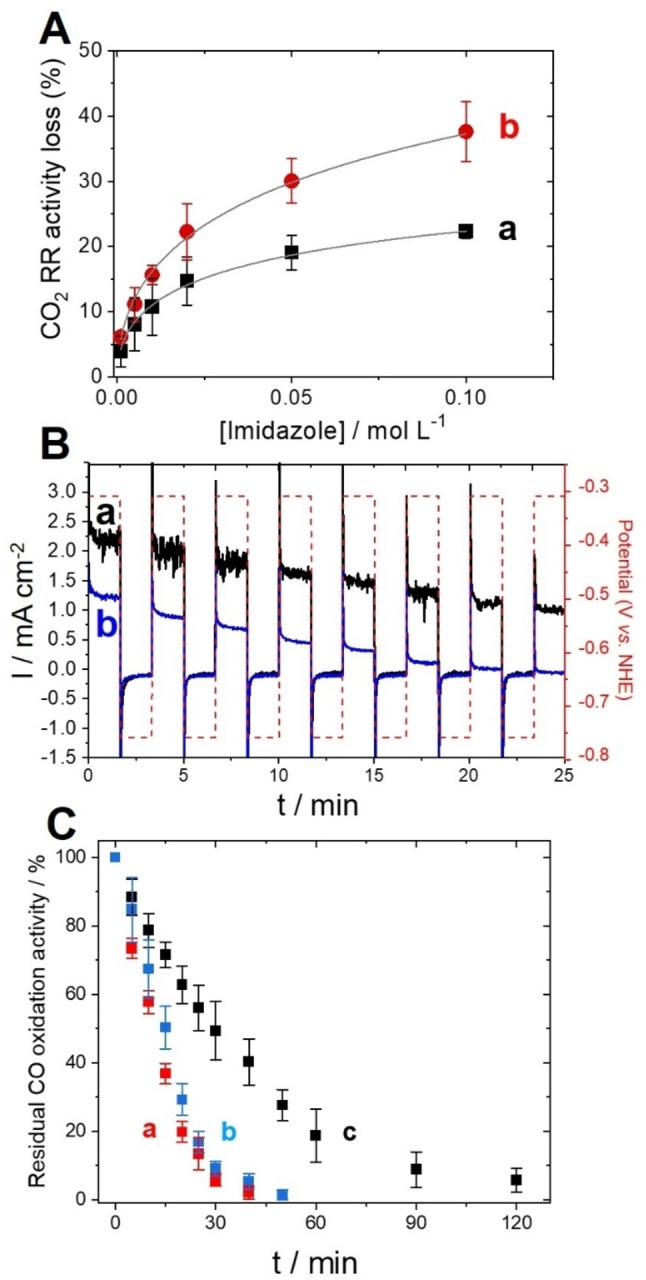
A) Plot of the CO_2_RR activity loss towards incubation (5 min) of imidazole with AcPyTACN‐modified MWCNT electrodes with (▪) Rec‐*Rr*CODH^His^ and (•) Rec‐*Rr*CODH (under CO_2_, 50 mM Tris‐HCl, pH 8.5, *v*=5 mV s^−1^) and corresponding simulated curves using a Langmuir–Freundlich isotherm model (gray line, see Supporting Information for details). B) Chronoamerometry performed at *E*_p_=−0.31 V and −0.76 V vs. NHE (right Y axis) in CO‐saturated pH 8.5 phosphate buffer for His‐tagged Rec‐*Rr*CODH immobilized at a) **AcPyTACN**‐modified MWCNT electrode and b) nonmodified electrode after increasing time exposure to air and reactivation at *E*_p_=−0.76 V for 100 seconds. C) Plot of the residual CO oxidation activity towards increasing time exposure to air for a) Rec‐*Rr*CODH^His^ in solution, b) Rec‐*Rr*CODH^His^ immobilized at non modified MWCNT electrode and c) Rec‐*Rr*CODH^His^ immobilized at **AcPyTACN**‐modified MWCNT electrode

In order to confirm the nature of the interactions between functionalized MWCNT and Rec‐*Rr*CODH^His^, bio‐functionalized MWCNT electrodes were incubated in the presence of increasing concentration of imidazole, a well‐known competing ligand towards Ni‐NTA‐histidine binding. As expected, the CO_2_RR is reduced after incubation with increasing concentrations of imidazole. A negligible decrease is observed if the electrodes are incubated without imidazole. Figure 4A displays the loss of CO_2_RR activity at pH 8.5 as a function of the incubating imidazole concentration. The curves exhibit a typical Langmuir‐type evolution towards imidazole concentration. Upon increasing concentration of imidazole, immobilized Rec‐*Rr*CODH^His^ is progressively replaced by imidazole at TACN sites. The curves follow an apparent Langmuir–Freundlich isotherm model (see Supporting Information for full details).[^45^, ^46^] The introduction of the Freundlich parameter is caused by the heterogeneity of the MWCNT surface towards imidazole binding. In the case of the His‐tagged enzyme, it is noteworthy that no complete loss of CO_2_RR activity is observed at high imidazole concentrations. The functionalized electrode exhibits only a 25 % decrease at 100 mM and a maximum decrease of 43 (+/−2) % according to the Langmuir–Freundlich isotherm (i.e. at infinite imidazole concentration). For the tag‐free enzyme, a decrease of 38 % is observed at 100 mM of imidazole and a maximum decrease of 94 (+/−11) is given by the model. Furthermore, low affinity constants of 4 (+/1) and 12 (+/−3) L mol^−1^, for immobilized Rec‐*Rr*CODH^His^ and Rec‐*Rr*CODH, respectively, confirm the strong attachment of the enzymes at the **AcPyTACN**‐functionalized electrode. The high stability of the Ni‐**AcPyTACN**‐functionalized MWCNT electrode towards imidazole might arise from the possible binding of the Rec‐*Rr*CODH^His^ to the coordinated Ni(II) centres via at least four histidine groups per dimer in the best conditions, as depicted in figure 3A. Ni‐**AcPyTACN** provides a homogeneous attachment of enzymes as has already been observed for the immobilization of small proteins on TACN‐based SAM on gold electrodes.^[33]^

After confirming the immobilization of Rec‐*Rr*CODH^His^ at Ni‐**AcPyTACN**‐functionalized MWCNT electrodes, we investigated the oxygen tolerance of immobilized CODH at these functionalized electrodes. First, the electrocatalytic CO_2_RR for Ni‐**AcPyTACN**‐functionalized MWCNT electrodes and nonmodified electrodes were compared towards increasing exposure time to air.Figure 4B displays the remaining electrocatalytic activity measured by chronoamperometry under CO performed at −0.31 V vs. NHE. After each exposure to air, a reduction step is performed at −0.76 V for 100 s in order to reactivate the enzyme towards CO oxidation. Experiments were performed on CO oxidation in order to compare electrocatalytic CO oxidation with CO oxidation measured in solution in the presence of methyl viologen as final electron acceptor. In solution, the enzyme is pre‐reduced and reactivated using dithionite^[16]^ (see Supporting Information for details). As it has been demonstrated before, irreversible deactivation is observed for CODH after exposure to air.[^18^, ^19^] Rec‐*Rr*CODH^His^ exhibits similar stability towards air exposure either in solution or immobilized on nonmodified MWCNTs retaining only 5 % of activity after 30 min. On the contrary, Rec‐*Rr*CODH^His^ still retains almost 10 % of its initial activity (220 μA cm^−2^) after 90 min exposure to air. These experiments show that the Ni‐**AcPyTACN** anchor not only provide stable immobilization of CODH but also improve oxygen tolerance of the enzyme. This likely arises from the high biocatalyst surface coverage offered by the nanostructured MWCNT, making substrate diffusion the limiting step of the catalysis. This is confirmed by the shape of the corresponding CV performed under CO (Figure S5) at increasing air exposure times. While first CVs exhibit a stable plateau corresponding to mass transport limitations, CVs performed after 60 min air exposure exhibit the typical shape of kinetically‐limited CO oxidation by immobilized CODHs.[^14^, ^15^] High potential conversion into C_o*x*_ state and reactivation at −0.25 V vs. NHE is observed. It is noteworthy that CO oxidation is also inhibited by the presence of oxygen in the solution. If an oxygen‐saturated buffer is added in the solution, complete deactivation of the electrocatalytic signal is observed when oxygen is added at a concentration of 178 μM. On the contrary, no inhibition is observed for CO_2_ reduction when similar amounts of oxygen are added (Figure S6). Although CODH has better affinity for CO than CO_2_ (*K*_m_ in the μM range vs. mM range), this is consistent with the fact that O_2_, such as other inhibitors of CODH (CN^−^ for instance), binds to the Cred1 state of the enzyme, likely at the Ni centre, and can be reversibly reactivated upon reduction.^[13]^ In light of these experiments performed under O_2_, it is difficult to link O_2_ resistance to a specific orientation of the enzyme owing to histidine binding. However, the high amount of enzymes immobilized via the AcPyTACN affords the bioelectrodes to achieve excellent stability over time owing to mass transport limitations.

**Figure 5:**
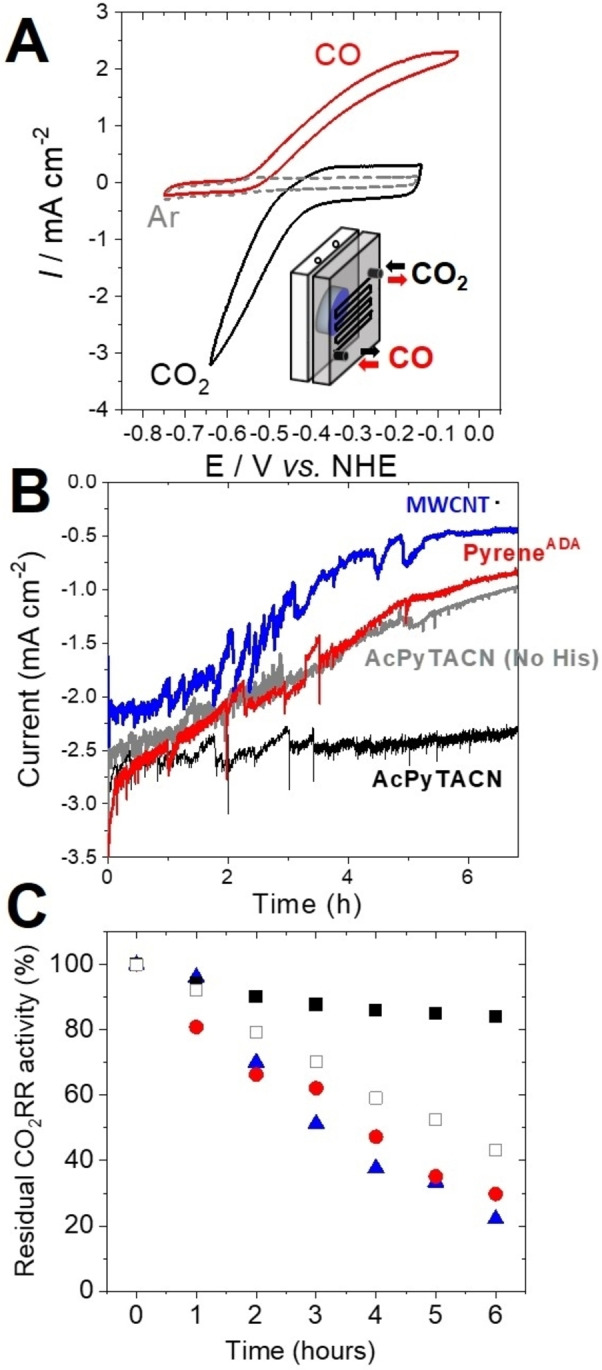
A) CVs of the Rec‐*Rr*CODH^His^‐functionalized Ni‐TACN‐modified gas‐diffusion bioelectrode under Ar, CO and CO_2_ (50 mM Tris‐HCl, pH 8.5, *v*=5 mV s^−1^). (inset) Schematic representation of the gas‐diffusion electrochemical cell. B) Chronoamperometry performed at −0.76 V vs. NHE under CO_2_ for 6 hours under CO_2_ for (grey) Rec‐*Rr*CODH^His^ immobilized at nonmodified MWCNT electrode, (red) Rec‐*Rr*CODH^His^ immobilized at pyrene^ADA^‐modified MWCNT electrode, (blue) Rec‐*Rr*CODH immobilized at **AcPyTACN**‐modified MWCNT electrode and (black) His‐tagged Rec‐RrCODH immobilized at **AcPyTACN**‐modified MWCNT electrode. C) Residual CO2RR activity measured each hour for (▴) Rec‐*Rr*CODH^His^ immobilized at nonmodified MWCNT electrode, (•) Rec‐*Rr*CODH^His^ immobilized at pyrene^ADA^‐modified MWCNT electrode, (□) Rec‐*Rr*CODH immobilized at **AcPyTACN**‐modified MWCNT electrode and (▪) Rec‐*Rr*CODH^His^ immobilized at **AcPyTACN**‐modified MWCNT electrode.

We recently developed a CO_2_‐diffusion bioelectrochemical cell where the enzyme is able to operate at a three‐phase boundary.^[16]^ This type of cell has also been developed for the reduction of CO_2_ into formate by formate dehydrogenase or the oxidation of H_2_ by hydrogenases.[^47^, ^48^, ^49^, ^50^, ^51^, ^52^] This cell was used to allow the integration of CODH at a gas‐diffusion electrode, ensuring efficient CO_2_ flux towards CODH, while maintaining the enzyme in its optimal buffer. Furthermore, no stirring of the solution is required and negligible acidification of the electrolyte is observed since CO_2_ is not directly purged in the electrolyte. No acidification of the buffer is observed along the course of hour‐long experiments under CO_2_. Figure 5A displays a typical CV performed at a gas‐diffusion electrode modified with Ni‐**AcPyTACN**‐functionalized MWCNT and Rec‐*Rr*CODH^His^ under Ar, CO and CO_2_ flux. A maximum current density of 3.2 mA cm^−2^ is obtained for electrocatalytic CO_2_ reduction at −0.65 V vs. NHE. A maximum current density of 2.2 mA cm^−2^ is obtained for CO oxidation at −0.1 V. It is noteworthy that current density is lower as compared to MWCNT electrodes in solution. In addition to the absence of stirring, this arises from a non‐optimized three‐phasic boundary where gas accessibility needs to be optimized at immobilized enzymes in contact with the electrolyte. CVs of different electrodes were performed under CO_2_ (Figure S7). The stability of these different type of electrodes was compared under constant applied potential for 6 hours, corresponding to an overpotential of 120 mV (Figure 5B). On nonmodified MWCNT electrodes, the CO_2_RR current density rapidly decreases over time, showing a residual activity of 22 % of the starting CO_2_RR current density after 6 hours. Despite higher enzyme surface coverage and starting current density, MWCNT electrodes modified with pyrene^ADA^ exhibit a residual activity of 29 %. Rec‐*Rr*CODH immobilized at **AcPyTACN**‐modified electrodes shows higher stability with residual activity of 43 %. For the immobilization of Rec‐*Rr*CODH^His^ at Ni‐**AcPyTACN**‐modified MWCNT electrodes, a small decrease is observed after 6 hours and a residual activity of 85 % is measured corresponding to a current density of 2.3 mA cm^−2^. This corresponds to a TON of 6×10^6^ and an average TOF of 300 s^−1^ over 6 hours. These results unambiguously demonstrate the high stability of the enzyme binding at these electrodes, outperforming stability performances in any other configurations. While the access to these catalysts are very distinct, these performances are in line with the best catalysts for CO_2_RR which are either based on highly efficient gold‐based nanomaterials^[53]^ or based on CNT‐supported molecular catalysts such as cobalt azamacrocycles.[^54^, ^55^, ^56^]

## Conclusion

This work shows the combination of a high‐performance CODH and an original pyrene anchor to achieve efficient and stable CO_2_ reduction at near‐zero overpotential with improved stability towards air exposure and negligible inhibition of oxygen during catalysis. The use of such original TACN‐based anchor molecules for the immobilization of high‐performance metalloenzymes opens new perspectives in enzyme immobilization for electrocatalytic applications. Next developments towards enzymatic CO_2_RR will aim at understanding and improving irreversible oxidative inactivation by this family of enzymes to improve their further integration in operational devices such as CO_2_ electrolyzers.

## Conflict of interest

The authors declare no conflict of interest.

1

## Supporting information

As a service to our authors and readers, this journal provides supporting information supplied by the authors. Such materials are peer reviewed and may be re‐organized for online delivery, but are not copy‐edited or typeset. Technical support issues arising from supporting information (other than missing files) should be addressed to the authors.

Supporting Information

## Data Availability

The data that support the findings of this study are available from the corresponding author upon reasonable request.
