# Supplementary material for: A Pyrene‐Triazacyclononane Anchor Affords High Operational Stability for CO2RR by a CNT‐Supported Histidine‐Tagged CODH
Source: Angew Chem Int Ed Engl. 2022 Mar 23;61(21):e202117212. doi: 10.1002/anie.202117212 (PMC9401053; doi:10.1002/anie.202117212)
Supplement: Supplementary file 1 — Supporting Information [file ANIE-61-0-s001.pdf]

Angewandte Chemie
Eine Zeitschrift der Gesellschaft Deutscher Chemiker

Supporting Information

A Pyrene-Triazacyclononane Anchor Affords High Operational Stability for CO_2RR by a CNT-Supported Histidine-Tagged CODH

U. Contaldo, M. Curtil, J. Pérard, C. Cavazza, A. Le Goff**

Supporting Information

Content:

1. Materials and methods……………………………………………….page 2

2. Synthesis of AcPyTACN………………………………………………..page 3

3. Preparation of electrodes…………………………………………page 4

4. ICP-AES metal ion analysis…………………………………………..page 6

5. Electrochemical Analysis……………………………………………..page 7

6. Figures and Tables……………………………………………………page 8

7. References…………………………………………………………….page 15

1. Materials and methods

Materials and Instruments. 1-pyrenebutyric acid adamantyl amide was prepared as previously described.[1] All reagents were purchased from Sigma Aldrich. Commercial grade thin Multi-Walled Carbon Nanotubes (MWCNT, 9.5 nm diameter, purity > 99%). Carbon nanomaterials were used as received without any purification. Rec*Rr*CODH was co-produced in presence of the three Ni-chaperones (*Rr*CooC, *Rr*CooT and *Rr*CooJ) and isolated as previously described[2]. When not used, the enzymes were stored at 4 °C. All the reagents were used without further purification. All solvents were of analytical grade. Distilled water was passed through a Milli-Q water purification system. Acetonitrile (HPLC) grade used for electrochemistry was obtained from VWR chemicals and used after drying on 4 Å molecular sieves. NMR spectra were recorded on a Bruker AM 300 (^{1}H at 300 MHz, ^{13}C at 75 MHz) or a Bruker Avance 400 (^{1}H at 400 MHz, ^{13}C at 100 MHz). Chemical shifts are given relative to solvent residual peak. Mass spectra were recorded on a Bruker Esquire 3000 (ESI/Ion Trap) equipment.

Electrochemical analysis. The electrochemical experiments in aqueous media were performed in 50 mM TrisHCl buffer pH 8.5 in a three-electrode electrochemical cell, using a Biologic VMP3 Multi Potentiostat, inside an anaerobic glove box (O_2 <2 ppm, Jacomex). The surface of GC electrodes was polished with a 2 µm diamond paste purchased from Presi (France) and rinsed successively with water, acetone, and ethanol. A Pt wire placed was used as counter electrode, and the SCE or Ag/AgCl served as reference electrodes. All current densities are given considering the geometrical surface of the MWCNT-modified electrode (0.07 cm^{-2}). Oxygen concentrations were measured in the electrolyte by using a Neofox Oxygen Sensing System from OceanOptics.

2. Synthesis of the ligands

Synthetic procedures for compound 1 and 2:

Addition of **1-Pyrenylmethyl bromide** (555mg, 1.87mmol) to a solution of tacn orthoamide (260 mg, 1.87 mmol) in tetrahydrofuran (6 ml) produced a precipitate almost immediately. Stirring was continued for another 30 minutes after which the product was filtered and washed with absolute ethanol (2 x 2 ml) and ether (3 x 2 ml). The green solid was then dissolved in water (6 mL) and heated at reflux for 4 hours. The solution pH was adjusted to 12 with NaOH, the product was extracted into chloroform (4 x 10 ml), the extracts dried over magnesium sulfate and the solvent removed under reduced pressure to give **1 (**411 mg, 87 % yield). **1** (411mg, 1.1 mmol) was then dissolved in acetonitrile (30 mL), sodium carbonate (3 g) and ethyl bromoacetate (252 µL, 2.3 mmol) were added. The mixture was stirred at reflux for 6 h. Solvents were removed under reduced pressure. The crude product was dissolved in a 5M aqueous solution of NaOH (pH=12) extracted into chloroform (3 x 10 ml). The gathered extracts were dried over magnesium sulfate and the solvent removed under reduced pressure to give a viscous, yellow/orange oil. The product was purified by column CH_2Cl_2/Acetone affording a white powder (255mg, 63% yield). ^{1}H NMR (400 MHz, $CDCl_3$), δ(ppm): 1.14 and 1.25 (3H, t, OEt, two resonance forms), 2.41 – 3.41(12H, m, CH_2 from macrocycle, two resonance forms),, 3.27 and 3.39 (2H, s, NCH_2COOEt, from two resonance forms), 4.05 and 4.15 (2H, OEt, two resonance forms), 4.32 and 4.33 (2H, s, CH_2-pyrene, from two resonance forms), 7.76-8.21 (9H, ms, CH from pyrene), 8.48 and 8.50 (1H, s, NCHO, from two resonance forms). ^{13}C NMR (100 MHz, $CDCl_3$) δ(ppm): 14.18 and 14.32 (CH3), 46.72 (CH2), 47.43 (CH2), 50.14(CH2), 51.12(CH2), 53.84(CH2), 53.94(CH2), 54.60(CH2), 54.80(CH2), 54.88(CH2), 55.56(CH2), 57.94(CH2), 58.08(CH2), 58.39(CH2), 58.47(CH2), 60.37(CH2), 60.43(CH2), 61.18(CH2), 61,71(CH2), 76,83(CH2), 77.09(CH2), 77.34(CH2), 124.04 (CH) 124.40 (CH), 124.42 (CH), 124.45 (CH), 124.76 (Cq), 124.82 (Cq), 125.01 (Cq), 125.08(CH), 125.10(CH), 125.18(CH), 125.33(CH), 125.91(CH), 126.01(CH), 127.09(CH), 127.16(CH), 127.29(CH), 127.40(CH), 127.49(CH), 128.13(CH), 128.31(CH), 129.81(Cq), 129.87(Cq), 130.88(Cq), 130.89(Cq), 130.93(Cq), 131.28(Cq), 131,33(Cq), 132.74(Cq), 133.16(Cq), 163.84 (CH), 171.78 (Cq), 172.10 (Cq)

Synthetic procedures for AcPyTACN:

Compound 2 (225m g 0.49 mmol) was dissolved in 5M HCl (5 ml) and the solution refluxed for 3h. Removal of the solvent gave the deprotected **AcPyTACN** as a green solid.

3. Preparation of the electrodes

The working electrodes were glassy carbon and gas diffusion electrodes (3 mm diameter). N-methyl-2-pyrrolidinone (NMP) dispersion of MWCNTs were prepared by 30 minutes sonication of 5 mg MWCNTs dispersed in 1 mL NMP until homogeneous black suspension was obtained. Then 20 µL of the MWCNTs solution were drop-casted on a GCE/GDE and NMP was removed under vacuum leaving a 5-µm-thick film on the GCE.

Functionalisation of MWCNT with AcPyTACN or 1-pyrenebutyric acid adamantyl amide and enzyme immobilization

MWCNT-modified electrodes were soaked for 30 minutes in DMF solution containing 10 mM 1-pyrenebutyric acid adamantyl amide and after rinsed in DMF solution and two times in MilliQ water. MWCNT-modified electrodes were soaked for 30 minutes in DMF solution containing 10 mM AcPyTACN and after rinsed in DMF solution and two times in MilliQ water. The AcPyTACN functionalised MWCNT electrodes were further soaked in 10 mM $NiCl_2$ (MilliQ) for 30 minutes and then rinsed two times in MilliQ water.

The functionalised electrodes were then incubated for 4 hours with 25 µL of the enzymatic His-tagged-Rec-*Rr*CODH and Rec-*Rr*CODH solutions (38 µM dimer concentration). The latter step was carried out inside an anaerobic glove box (O_2 <2 ppm, Jacomex). The electrodes were finally washed with buffer 50 mM Tris-HCl, pH 8.5. When not used the electrodes were kept in buffer 50 mM Tris-HCl, pH 8.5.

CO oxidation activity measurements after air exposure

CO oxidation activity was assayed at 25 °C by following the reduction of methyl viologen at 604 nm [ε=13.6 mM^{-1} cm^{-1}] in CO-saturated solutions containing 12 mM methyl viologen and 1 mM DTT. CODH specific activity unit is expressed as micromoles of CO oxidized per minute per

milligram of protein. CODH 115 µM (monomer) in 50 mM Tris-HCl pH 8.5, 5 mM DTT, 5 mM Dithionite (DTH) was diluted to 23 nM (monomer) in 50 mM TrisHCl pH 8.5, remaining 1 µM of DTT and DTH. Then, the enzyme was exposed to the air for several times (0-40 minutes) at 25 °C. The enzyme was further diluted to 5 nM (monomer) in anaerobic 50 mM TrisHCl pH 8.5, 5 mM dithiothreitol (DTT), 1 mM dithionite (DTH) and incubated 5 minutes in this buffer in order to pre-activate the enzyme before measuring the remaining specific activity.

4. ICP-AES metal ion analysis

MWCNT films of 1.8 cm^{-2} were modified according to the procedure described in previous section. Then, the modified MWCNT electrodes were mineralized in the presence of 0,6 mL HNO_3 (65%) at 60 °C for 24 h. In order to remove traces of carbon nanotubes, the solution is first centrifuged 10 min at 5000 rpm, filtered on a glass filter and washed with 10% nitric acid solution before completing the volume to 6ml with pure water. . The metal concentration of the supernatant was analyzed by inductively coupled plasma atomic emission spectroscopy (ICP-AES) (Shimadzu ICP 9000 with mini plasma torch in axial reading mode). Standard solutions of Ni and Fe for atomic absorption spectroscopy (Sigma Aldrich) were used for quantification (calibration curve between 1.9 and 1000 µg L^{-1} in 10% HNO_3 (Fluka)). The results are presented in Table S1

Table S1. ICP-AES metal ion analysis (Concentration in µM in the supernatants)

**Rec-RrCODH^{His}-functionalized MWCNT electrode**	**Ni (µM)**	**Fe (µM)**
**Ni-AcPyTACN-modified electrodes**	240 (+/-20)	214 (+/-15)
**pristine MWCNT electrode**	25 (+/-5)	158 (+/-20)
**Control (MWCNT only)**	4 (+/-2)	16 (+/-2)

5. Electrochemical analysis

The Langmuir-Freundlich model was employed to fit the experimental data using OriginPro 2020, according to equation 1:[3,4]

$$\%Loss_{eq} = \frac{\%loss_{max} \times (K_{Imid}^{app} \times [\text{imidazole}])^n}{1 + (K_{Imid}^{app} \times [\text{imidazole}])^n}$$

Where $\%Loss_{eq}$ is the percentage of CO activity loss at the equilibrium, $\%loss_{max}$ is the maximum percentage of CO activity loss at maximum imidazole concentration, K_{Imid}^{app} is the apparent association constant in water between imidazole and AcPyTACN sites at the modified electrode and n is Langmuir-Freundlich coefficient number. Table S2 shows the Langmuir-Freundlich model parameters obtained from fitting curves from Figure 4A.

Table S2. Fitting parameters for the Langmuir-Freundlich isotherm model from figure 4A.

	**$\%Loss_{max}$**	**K_{Imid}^{app} (L mol^{-1})**	**n**	**R^2**
**Rec-*Rr*CODH**	94 (+/-12)	4 (+/-) 1	0.5	0.995
**Rec-*Rr*CODH^{His}**	43 (+/-2)	12 (+/-3)	0.5	0.999

5. Figures

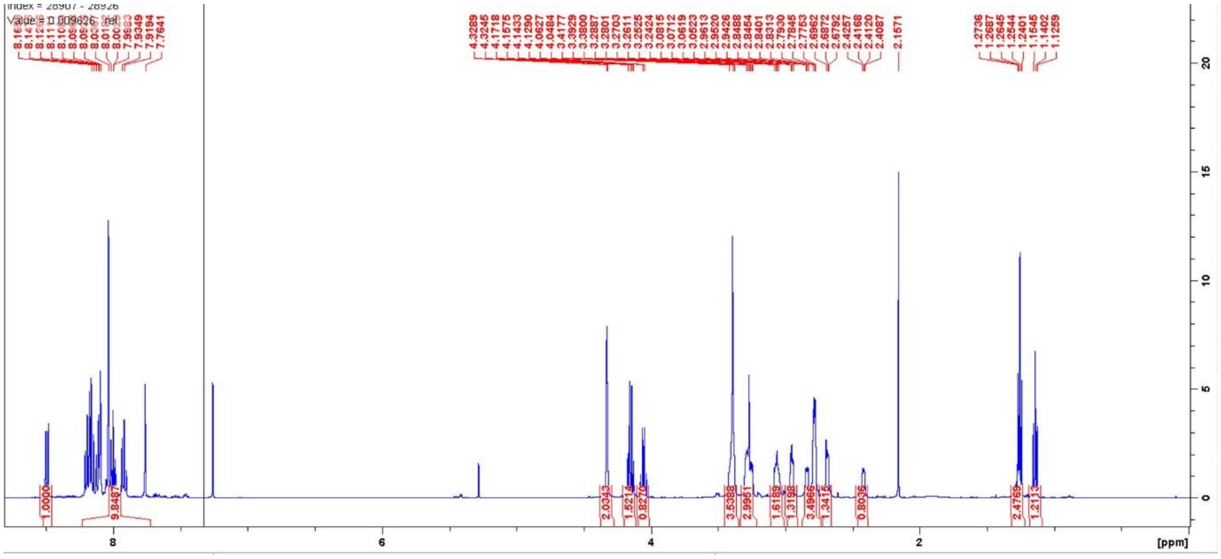

Figure S1. ^{1}HNMR spectrum of compound 2 in $CDCl_3$

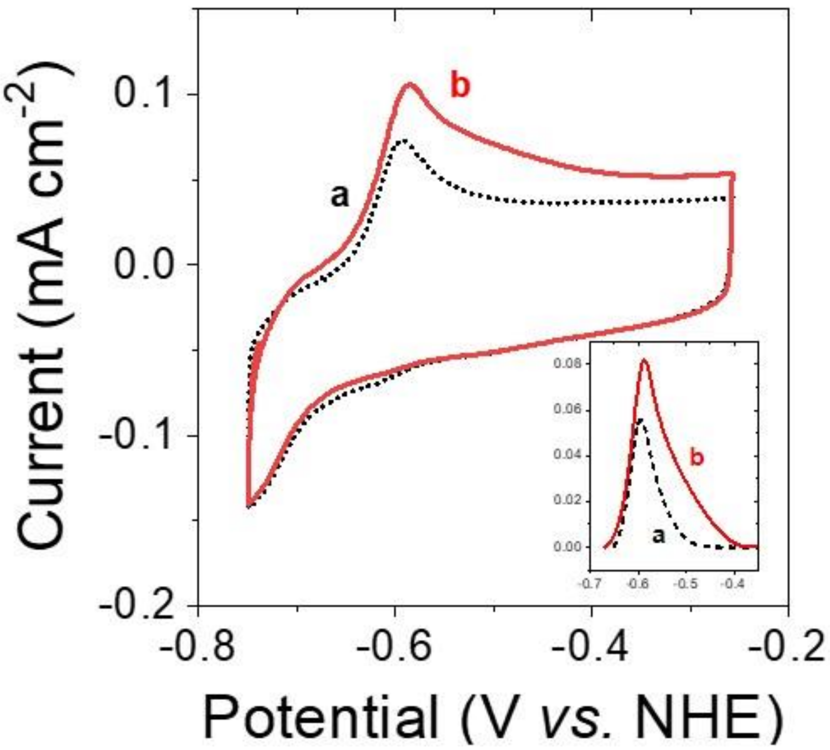

Figure S2. CVs and corresponding background-substracted anodic part (inset) of the Rec-*Rr*CODH-functionalized (a, black) pristine MWCNT electrode and (b, red) Ni-AcPyTACN-modified MWCNT electrodes under Ar after addition of 2 mM sodium cyanide (50 mM Tris-HCl, pH 8.5, v = 5 mV s^{-1}).

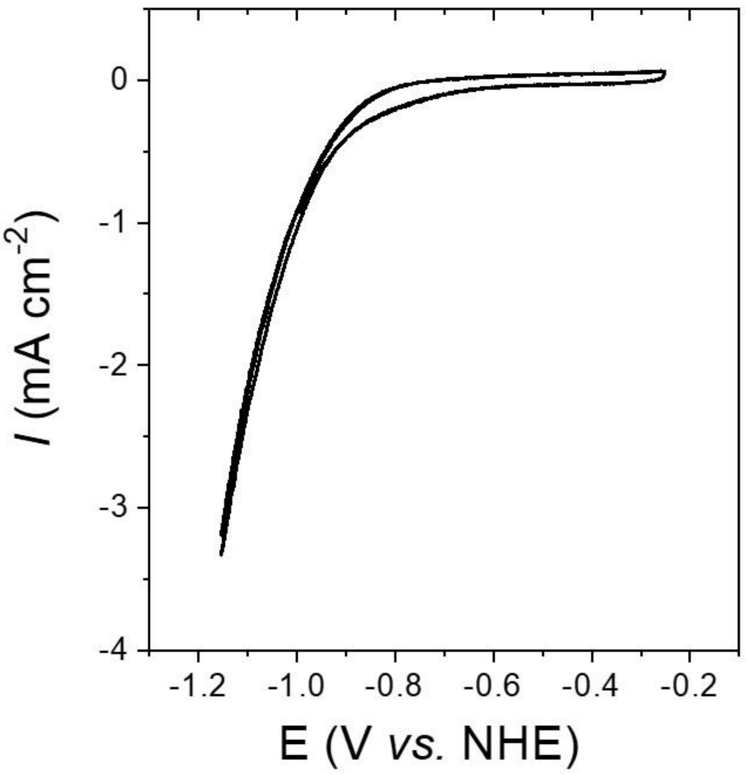

Figure S3. CVs of the MWCNT electrode modified with Ni-AcPyTACN under CO_2 (50 mM Tris-HCl, pH 8.5, v = 5 mV s^{-1}). The catalytic current at low potential corresponds to proton reduction by MWCNT electrode.

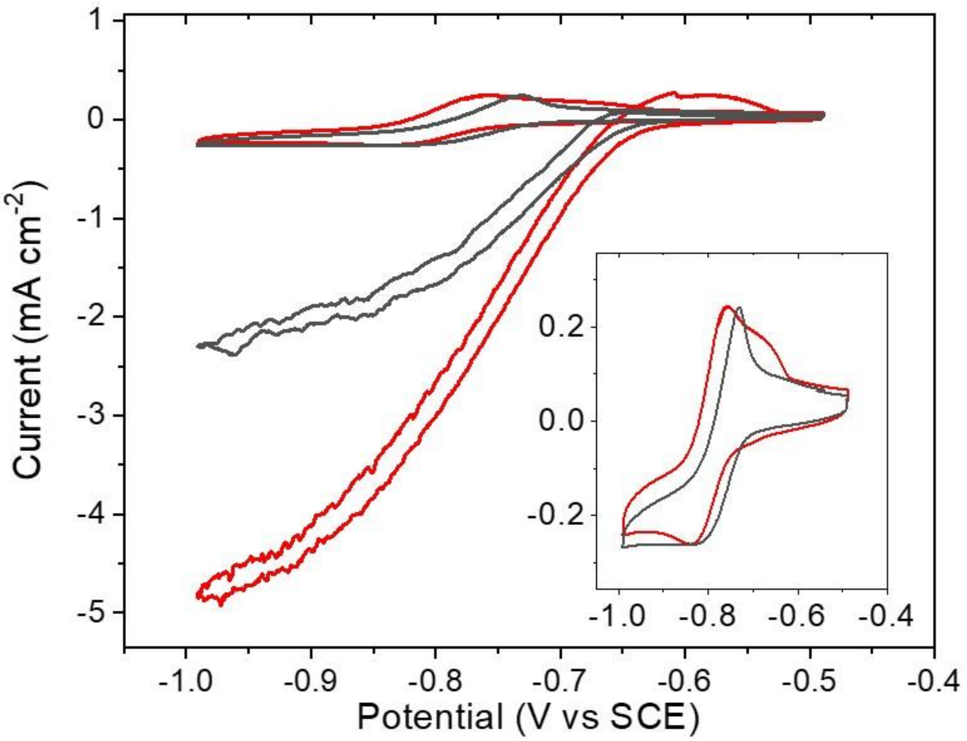

Figure S4. CVs of the Rec-*Rr*CODH-functionalized (black) pristine MWCNT electrode and (red) Ni-AcPyTACN-modified MWCNT electrodes under Ar (inset) and CO_2 (50 mM Tris-HCl, pH 8.5, v = 5 mV s^{-1}).

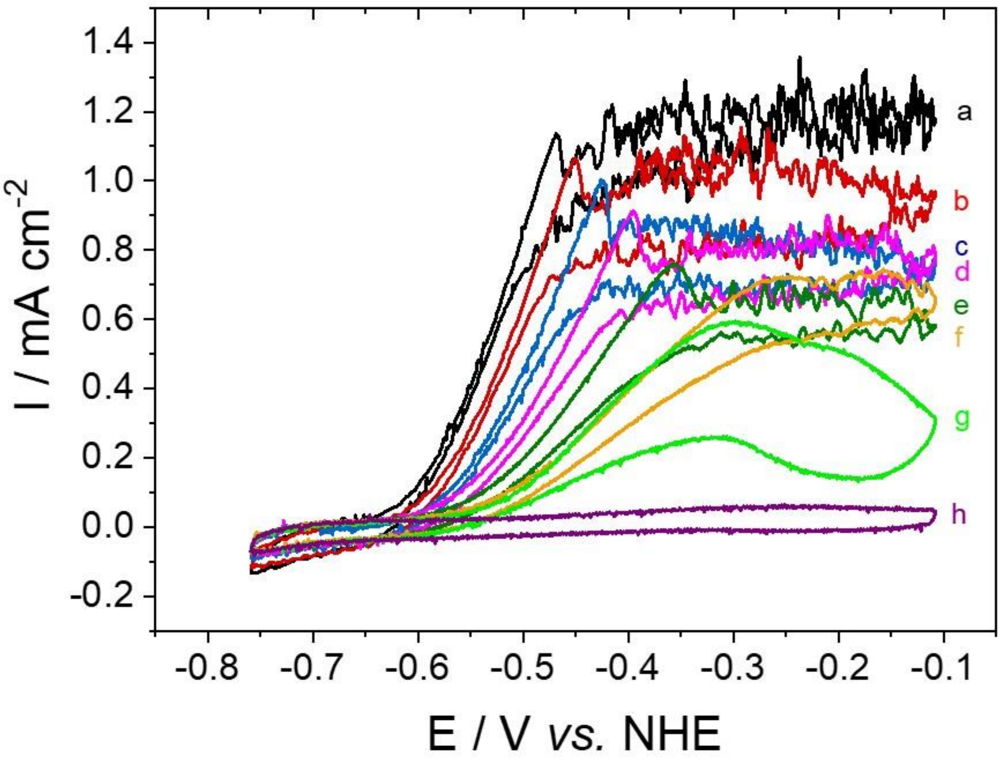

Figure S5. CVs of the His-tagged Rec-*Rr*CODH immobilized at Ni-AcPyTACN-modified MWCNT electrode under CO after increasing time exposure to air (a: 0, b: 10, c: 20, d: 30, e: 40, f: 50, g: 60, h: 120 min) and reactivation at Ep = -0.78 V for 100 seconds (50 mM Tris-HCl, pH 8.5, v = 5 mV s^{-1})

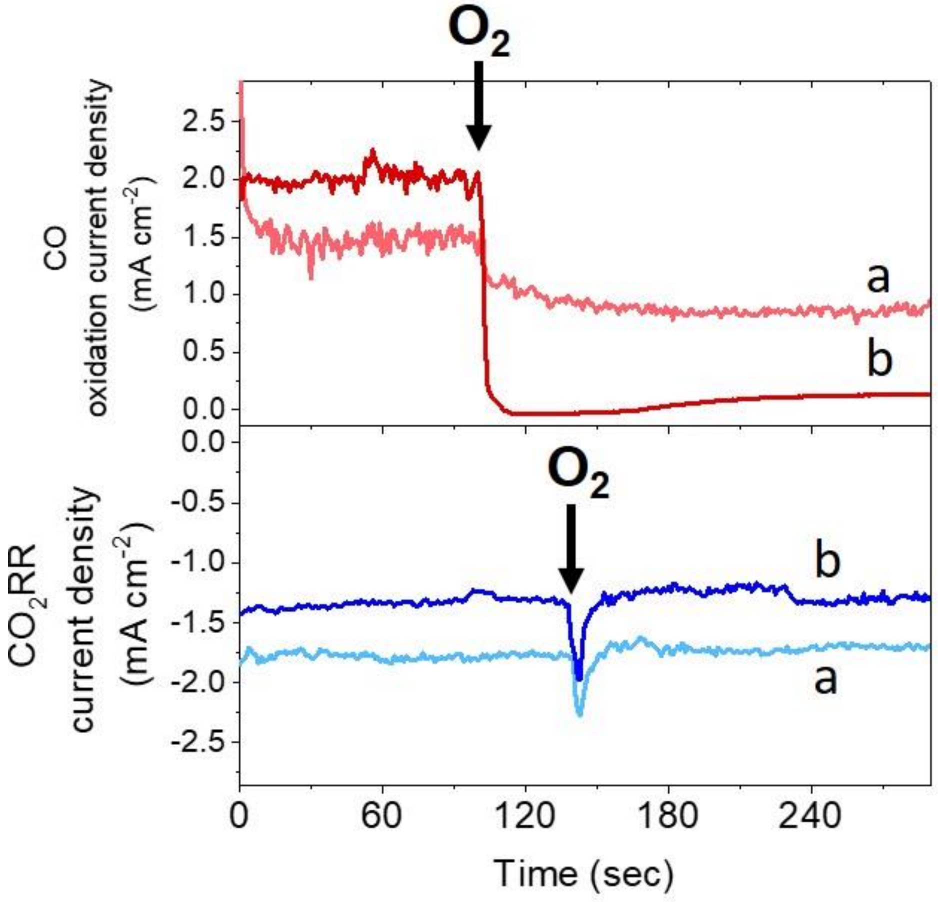

Figure S6. Chronoamerometry of the His-tagged Rec-*Rr*CODH immobilized at Ni-AcPyTACN-modified MWCNT electrode. Performed at Ep = -0.009 V *vs.* SHE in CO-saturated 50 mM Tris-HCl, pH 8.5 (UP) after addition of (a) 95 and (b) 178 µM O_2. (down) Chronoamerometry performed at Ep = -0.76 V *vs.* SHE in CO_2-saturated pH 8.5 phosphate buffer for his-tagged Rec-*Rr*CODH immobilized at Ni-AcPyTACN-modified MWCNT electrode after addition of (a) 105 and (b) 178 µM O_2 (50 mM Tris-HCl, pH 8.5). Final O_2 concentration was measured *in situ* using an optical probe (NEOFOX).

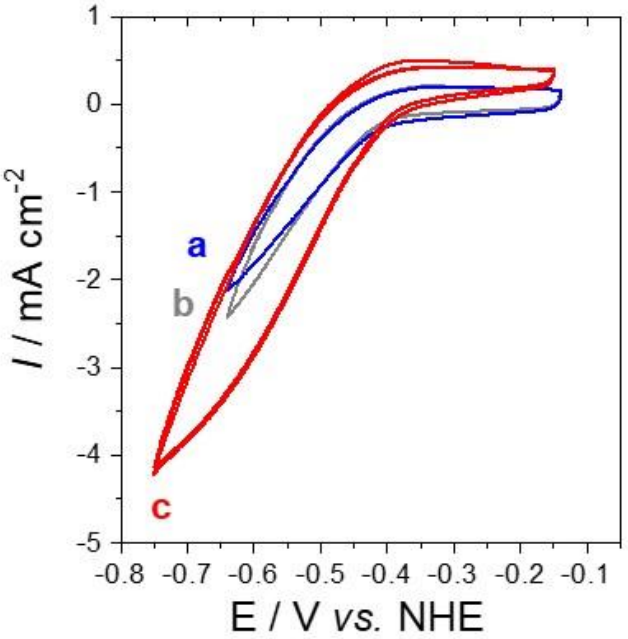

Figure S7. CVs of the (a) Rec-*Rr*CODH^{His} immobilized at nonmodified MWCNT electrode, (b) Rec-*Rr*CODH immobilized at Ni-AcPyTACN-modified MWCNT electrode and (c) Rec-*Rr*CODH^{His} immobilized at pyrene^{ADA}-modified MWCNT electrode (50 mM Tris-HCl, pH 8.5, v = 5 mV s^{-1}).

5. References

[1] R. Haddad, M. Holzinger, R. Villalonga, A. Neumann, J. Roots, A. Maaref, S. Cosnier, *Carbon* **2011**, *49*, 2571–2578.

[2] U. Contaldo, B. Guigliarelli, J. Perard, C. Rinaldi, A. Le Goff, C. Cavazza, *ACS Catal.* **2021**, 5808–5817.

[3] F. Haddache, A. Le Goff, B. Reuillard, K. Gorgy, C. Gondran, N. Spinelli, E. Defrancq, S. Cosnier, *Chem. Eur. J.* **2014**, *20*, 15555–15560.

[4] S. Sharma, G. P. Agarwal, *Anal. Biochem.* **2001**, *288*, 126–140.
